# 5-Methyl-*N*-[2-(trifluoro­meth­yl)phen­yl]isoxazole-4-carboxamide

**DOI:** 10.1107/S1600536812016467

**Published:** 2012-04-21

**Authors:** De-Cai Wang, Jiang-Kai Qiu, Hai-Xi Zhu, Ping Wei, Ping-Kai Ou-Yang

**Affiliations:** a State Key Laboratory of Materials-Oriented Chemical Engineering, School of Pharmaceutical Sciences, Nanjing University of Technology, Xinmofan Road No.5 Nanjing, Nanjing 210009, People’s Republic of China

## Abstract

In the title compound, C_12_H_9_F_3_N_2_O_2_, the benzene ring is nearly perpendicular to the isoxazole ring, making a dihedral angle of 82.97 (2)°. In the crystal, mol­ecules are linked by N—H⋯O hydrogen bonds into a supra­molecular chain running along the *c* axis.

## Related literature

For applications of leflunomide [systematic name: 5-methyl-*N*-[4-(trifluoro­meth­yl) phen­yl]-isoxazole-4-carboxamide] in the treatment of rheumatoid arthritis, see: Shaw *et al.* (2011 ▶); Schattenkirchner (2000 ▶). For leflunomide analogs, see: Huang *et al.* (2003 ▶); Wang *et al.* (2011 ▶).
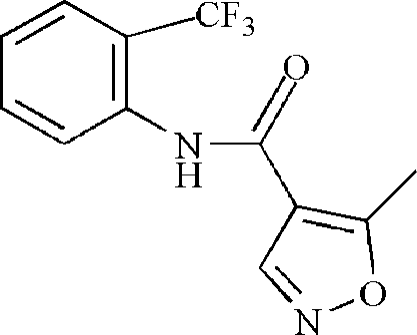


## Experimental

### 

#### Crystal data

C_12_H_9_F_3_N_2_O_2_
*M* *_r_* = 270.21
Monoclinic, 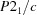

*a* = 15.839 (3) Å
*b* = 8.3260 (17) Å
*c* = 9.4250 (19) Å
β = 101.29 (3)°
*V* = 1218.9 (4) Å^3^
*Z* = 4
Mo *K*α radiation
μ = 0.13 mm^−1^
*T* = 293 K
0.20 × 0.10 × 0.10 mm

#### Data collection

Enref–Nonius CAD-4 diffractometer
Absorption correction: ψ scan (North *et al.*, 1968 ▶) *T*_min_ = 0.974, *T*_max_ = 0.987
2193 measured reflections
2193 independent reflections
1125 reflections with *I* > 2σ(*I*)
3 standard reflections every 200 reflections intensity decay: 1%

#### Refinement

*R*[*F* ^2^ > 2σ(*F* ^2^)] = 0.062
*wR*(*F* ^2^) = 0.164
*S* = 1.00
2193 reflections
172 parameters
H-atom parameters constrained
Δρ_max_ = 0.19 e Å^−3^
Δρ_min_ = −0.19 e Å^−3^

### 

Data collection: *CAD-4 EXPRESS* (Enraf–Nonius, 1994 ▶); cell refinement: *CAD-4 EXPRESS*; data reduction: *XCAD4* (Harms & Wocadlo,1995 ▶); program(s) used to solve structure: *SHELXTL* (Sheldrick, 2008 ▶); program(s) used to refine structure: *SHELXTL*; molecular graphics: *SHELXTL*; software used to prepare material for publication: *SHELXTL*.

## Supplementary Material

Crystal structure: contains datablock(s) I, global. DOI: 10.1107/S1600536812016467/xu5516sup1.cif

Structure factors: contains datablock(s) I. DOI: 10.1107/S1600536812016467/xu5516Isup2.hkl

Supplementary material file. DOI: 10.1107/S1600536812016467/xu5516Isup3.cml

Additional supplementary materials:  crystallographic information; 3D view; checkCIF report

## Figures and Tables

**Table 1:** Hydrogen-bond geometry (Å, °)

*D*—H⋯*A*	*D*—H	H⋯*A*	*D*⋯*A*	*D*—H⋯*A*
N1—H1*A*⋯O1^i^	0.86	2.13	2.855 (3)	142

Symmetry code: (i) 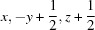.

**Fig. 1.**
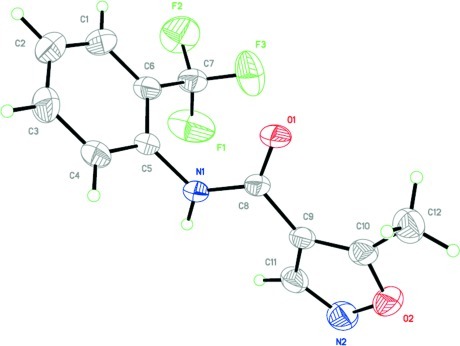
The structure of the title compound, showing the atomic numbering scheme. Non-H atoms are shown with 30% probability displacement ellipsoids.

## supplementary crystallographic information

### Comment

Leflunomide is one of the most effective isoxazole-containing disease-modifying drugs for treating rheumatoid arthritis (Shaw *et al.*, 2011; Schattenkirchner, 2000). Many leflunomide analogs have been synthesized and exhibit potent immunomodulating effect (Huang, *et al.*, 2003). In our previous work, some anolog has been sucessfully sythesized (Wang *et al.*, 2011). In this paper, one new leflunomide analog N-(2,4-difluorophenyl)-5-methylisoxazole-4-carboxamide monohydrate, was synthesized as a novel and potent immunomodulating drugs. We report herein its crystal structure.

As illustrated in Fig. 1, the molecular structure of the title compound is not planar. The C1-C6 benzene and the C9-C11/N2/O2 isoxazole ring is almost perpendicular to each other with the dihedral angle of 82.97 (2) °. The central nitrogen atom (N1) and carbon atom (C8) are nearly coplanar with the benzene ring and the isoxazole rings [N1-C5-C6-C1 torsion angles = -176.7 (3) ° and C8-C9-C10-O2 torsion angles = -177.9 (3) °], respectively. The length of the C11=N2 double bond is 1.295 (5) Å, slightly longer than standard 1.28 Å value of a C=N double bond. The crystal structure is stabilized by N—H···O hydrogen bonds(Table 1).

### Experimental

A solution of 0.05 mole of 5-methylisoxazole-4-carboxylic acid chloride (7.3 g) in 20 ml of acetonitrile is added drop-wise, while stirring, to 0.1 mole of 2-(trifluoromethyl)aniline (16.1g),dissolved in 150 ml of acetonitrile at room temperature.After stirring for 40 minutes, the precipitated 2-(trifluoromethyl)aniline hydrochloride is filtered off and washed with 100 ml portions of acetonitrile, and the combined filtrates are concentrated under reduced pressure.9.6g(69.51% of theory) of white crystalline 5-methyl-N-(2-(trifluoromethyl)phenyl)isoxazole-4- carboxamide are thus obtained. Crystals of (I) suitable for X-ray diffraction were obtained by slow evaporation of an methylbenzene solution.

### Refinement

H atoms were placed at calculated positions and were treated as riding on the parent C or N atoms with C—H = 0.96 (methyl), 0.97 (methylene) and N—H = 0.86 Å, *U*_iso_(H) = 1.2 or 1.5 *U*_eq_(C,N).

### Crystal data

**Table:**

C_12_H_9_F_3_N_2_O_2_	*F*(000) = 552
*M**_r_* = 270.21	*D*_x_ = 1.472 Mg m^−^^3^
Monoclinic, *P*2_1_/*c*	Mo *K*α radiation, λ = 0.71073 Å
Hall symbol: -P 2ybc	Cell parameters from 25 reflections
*a* = 15.839 (3) Å	θ = 9–13°
*b* = 8.3260 (17) Å	µ = 0.13 mm^−^^1^
*c* = 9.4250 (19) Å	*T* = 293 K
β = 101.29 (3)°	Block, colorless
*V* = 1218.9 (4) Å^3^	0.20 × 0.10 × 0.10 mm
*Z* = 4	

### Data collection

**Table:**

Enref–Nonius CAD-4 diffractometer	1125 reflections with *I* > 2σ(*I*)
Radiation source: fine-focus sealed tube	*R*_int_ = 0.000
Graphite monochromator	θ_max_ = 25.2°, θ_min_ = 1.3°
ω/2θ scans	*h* = −18→18
Absorption correction: ψ scan (North *et al.*, 1968)	*k* = 0→9
*T*_min_ = 0.974, *T*_max_ = 0.987	*l* = 0→11
2193 measured reflections	3 standard reflections every 200 reflections
2193 independent reflections	intensity decay: 1%

### Refinement

**Table:**

Refinement on *F*^2^	Primary atom site location: structure-invariant direct methods
Least-squares matrix: full	Secondary atom site location: difference Fourier map
*R*[*F*^2^ > 2σ(*F*^2^)] = 0.062	Hydrogen site location: inferred from neighbouring sites
*wR*(*F*^2^) = 0.164	H-atom parameters constrained
*S* = 1.00	*w* = 1/[σ^2^(*F*_o_^2^) + (0.068*P*)^2^] where *P* = (*F*_o_^2^ + 2*F*_c_^2^)/3
2193 reflections	(Δ/σ)_max_ < 0.001
172 parameters	Δρ_max_ = 0.19 e Å^−^^3^
0 restraints	Δρ_min_ = −0.19 e Å^−^^3^

### Special details

**Table:**

Geometry. All esds (except the esd in the dihedral angle between two l.s. planes) are estimated using the full covariance matrix. The cell esds are taken into account individually in the estimation of esds in distances, angles and torsion angles; correlations between esds in cell parameters are only used when they are defined by crystal symmetry. An approximate (isotropic) treatment of cell esds is used for estimating esds involving l.s. planes.
Refinement. Refinement of F^2^ against ALL reflections. The weighted R-factor wR and goodness of fit S are based on F^2^, conventional R-factors R are based on F, with F set to zero for negative F^2^. The threshold expression of F^2^ > 2sigma(F^2^) is used only for calculating R-factors(gt) etc. and is not relevant to the choice of reflections for refinement. R-factors based on F^2^ are statistically about twice as large as those based on F, and R- factors based on ALL data will be even larger.

### Fractional atomic coordinates and isotropic or equivalent isotropic displacement parameters (Å^2^)

**Table:**

	*x*	*y*	*z*	*U*_iso_*/*U*_eq_	
N1	0.73554 (18)	0.2300 (3)	0.9712 (3)	0.0540 (8)	
H1A	0.7386	0.2533	1.0610	0.065*	
O1	0.67807 (16)	0.2925 (3)	0.7394 (2)	0.0647 (8)	
F1	0.9108 (2)	0.3541 (4)	1.0116 (3)	0.1322 (13)	
C1	0.9018 (3)	−0.0106 (6)	0.8447 (4)	0.0732 (12)	
H1B	0.9526	0.0039	0.8108	0.088*	
O2	0.55089 (19)	0.6534 (3)	0.9342 (3)	0.0782 (9)	
N2	0.6083 (3)	0.6480 (4)	1.0694 (4)	0.0790 (11)	
F2	0.97802 (18)	0.2773 (4)	0.8551 (4)	0.1233 (11)	
C2	0.8701 (3)	−0.1615 (6)	0.8525 (5)	0.0844 (14)	
H2B	0.8989	−0.2489	0.8232	0.101*	
F3	0.85728 (19)	0.3827 (3)	0.7915 (3)	0.1128 (10)	
C3	0.7957 (3)	−0.1853 (5)	0.9035 (5)	0.0805 (13)	
H3A	0.7746	−0.2886	0.9103	0.097*	
C4	0.7526 (3)	−0.0551 (5)	0.9447 (4)	0.0650 (11)	
H4A	0.7023	−0.0707	0.9797	0.078*	
C5	0.7837 (2)	0.0983 (4)	0.9344 (3)	0.0512 (9)	
C6	0.8601 (2)	0.1205 (5)	0.8858 (4)	0.0574 (10)	
C7	0.9005 (3)	0.2832 (5)	0.8849 (5)	0.0680 (11)	
C8	0.6852 (2)	0.3198 (4)	0.8696 (4)	0.0486 (9)	
C9	0.6396 (2)	0.4541 (4)	0.9236 (4)	0.0488 (9)	
C10	0.5722 (3)	0.5371 (5)	0.8487 (4)	0.0589 (10)	
C11	0.6589 (3)	0.5281 (5)	1.0597 (4)	0.0641 (11)	
H11A	0.7029	0.4948	1.1343	0.077*	
C12	0.5197 (3)	0.5268 (5)	0.7026 (4)	0.0761 (12)	
H12A	0.4770	0.6101	0.6897	0.114*	
H12B	0.4919	0.4240	0.6897	0.114*	
H12C	0.5559	0.5397	0.6327	0.114*	

### Atomic displacement parameters (Å^2^)

**Table:**

	*U*^11^	*U*^22^	*U*^33^	*U*^12^	*U*^13^	*U*^23^
N1	0.066 (2)	0.0617 (19)	0.0402 (15)	0.0153 (17)	0.0235 (14)	0.0065 (15)
O1	0.0783 (18)	0.0810 (19)	0.0400 (13)	0.0171 (15)	0.0239 (12)	−0.0014 (13)
F1	0.172 (3)	0.127 (3)	0.112 (2)	−0.071 (2)	0.062 (2)	−0.0454 (19)
C1	0.064 (3)	0.081 (3)	0.083 (3)	0.013 (2)	0.035 (2)	0.003 (2)
O2	0.093 (2)	0.0569 (17)	0.094 (2)	0.0188 (16)	0.0402 (19)	0.0020 (16)
N2	0.111 (3)	0.063 (2)	0.069 (2)	0.010 (2)	0.035 (2)	−0.0060 (19)
F2	0.0791 (18)	0.124 (2)	0.182 (3)	−0.0192 (18)	0.0619 (19)	0.010 (2)
C2	0.091 (3)	0.071 (3)	0.100 (4)	0.022 (3)	0.040 (3)	0.003 (3)
F3	0.108 (2)	0.087 (2)	0.135 (3)	−0.0171 (17)	0.0032 (18)	0.0453 (18)
C3	0.097 (3)	0.049 (3)	0.102 (3)	0.003 (2)	0.034 (3)	0.014 (2)
C4	0.065 (3)	0.068 (3)	0.070 (3)	−0.001 (2)	0.033 (2)	0.015 (2)
C5	0.059 (2)	0.054 (2)	0.045 (2)	0.0066 (19)	0.0234 (18)	0.0067 (16)
C6	0.058 (2)	0.069 (3)	0.050 (2)	0.006 (2)	0.0218 (18)	0.0051 (19)
C7	0.065 (3)	0.075 (3)	0.071 (3)	0.000 (2)	0.033 (2)	0.004 (2)
C8	0.050 (2)	0.049 (2)	0.052 (2)	0.0024 (18)	0.0254 (17)	0.0047 (17)
C9	0.061 (2)	0.044 (2)	0.0470 (19)	−0.0004 (18)	0.0261 (17)	0.0010 (17)
C10	0.064 (3)	0.049 (2)	0.070 (2)	−0.004 (2)	0.030 (2)	0.004 (2)
C11	0.091 (3)	0.056 (2)	0.051 (2)	0.003 (2)	0.026 (2)	0.0006 (19)
C12	0.076 (3)	0.072 (3)	0.081 (3)	−0.001 (2)	0.017 (2)	0.015 (2)

### Geometric parameters (Å, º)

**Table:**

N1—C8	1.346 (4)	C3—C4	1.377 (5)
N1—C5	1.417 (4)	C3—H3A	0.9300
N1—H1A	0.8600	C4—C5	1.379 (5)
O1—C8	1.231 (4)	C4—H4A	0.9300
F1—C7	1.314 (4)	C5—C6	1.387 (4)
C1—C2	1.361 (6)	C6—C7	1.499 (5)
C1—C6	1.370 (5)	C8—C9	1.475 (4)
C1—H1B	0.9300	C9—C10	1.350 (5)
O2—C10	1.345 (4)	C9—C11	1.401 (5)
O2—N2	1.415 (4)	C10—C12	1.465 (5)
N2—C11	1.295 (5)	C11—H11A	0.9300
F2—C7	1.312 (4)	C12—H12A	0.9600
C2—C3	1.370 (6)	C12—H12B	0.9600
C2—H2B	0.9300	C12—H12C	0.9600
F3—C7	1.302 (5)		
			
C8—N1—C5	121.8 (3)	F3—C7—F1	106.5 (4)
C8—N1—H1A	119.1	F2—C7—F1	104.9 (4)
C5—N1—H1A	119.1	F3—C7—C6	114.2 (4)
C2—C1—C6	121.1 (4)	F2—C7—C6	112.7 (4)
C2—C1—H1B	119.5	F1—C7—C6	112.2 (3)
C6—C1—H1B	119.5	O1—C8—N1	122.2 (3)
C10—O2—N2	108.9 (3)	O1—C8—C9	121.8 (3)
C11—N2—O2	105.0 (3)	N1—C8—C9	115.9 (3)
C1—C2—C3	120.3 (4)	C10—C9—C11	105.2 (3)
C1—C2—H2B	119.9	C10—C9—C8	126.8 (3)
C3—C2—H2B	119.9	C11—C9—C8	128.0 (4)
C2—C3—C4	119.5 (4)	O2—C10—C9	108.7 (3)
C2—C3—H3A	120.3	O2—C10—C12	116.3 (4)
C4—C3—H3A	120.3	C9—C10—C12	135.0 (4)
C3—C4—C5	120.4 (3)	N2—C11—C9	112.2 (4)
C3—C4—H4A	119.8	N2—C11—H11A	123.9
C5—C4—H4A	119.8	C9—C11—H11A	123.9
C4—C5—C6	119.5 (3)	C10—C12—H12A	109.5
C4—C5—N1	118.9 (3)	C10—C12—H12B	109.5
C6—C5—N1	121.6 (3)	H12A—C12—H12B	109.5
C1—C6—C5	119.2 (4)	C10—C12—H12C	109.5
C1—C6—C7	119.2 (3)	H12A—C12—H12C	109.5
C5—C6—C7	121.6 (3)	H12B—C12—H12C	109.5
F3—C7—F2	105.6 (3)		
			
C10—O2—N2—C11	−1.6 (4)	C1—C6—C7—F1	−123.8 (4)
C6—C1—C2—C3	−0.7 (7)	C5—C6—C7—F1	52.7 (5)
C1—C2—C3—C4	1.0 (7)	C5—N1—C8—O1	0.1 (5)
C2—C3—C4—C5	0.2 (6)	C5—N1—C8—C9	−179.3 (3)
C3—C4—C5—C6	−1.7 (6)	O1—C8—C9—C10	16.6 (5)
C3—C4—C5—N1	177.1 (4)	N1—C8—C9—C10	−163.9 (3)
C8—N1—C5—C4	−100.9 (4)	O1—C8—C9—C11	−160.1 (3)
C8—N1—C5—C6	77.9 (4)	N1—C8—C9—C11	19.4 (5)
C2—C1—C6—C5	−0.8 (6)	N2—O2—C10—C9	1.4 (4)
C2—C1—C6—C7	175.8 (4)	N2—O2—C10—C12	−179.2 (3)
C4—C5—C6—C1	2.0 (5)	C11—C9—C10—O2	−0.7 (4)
N1—C5—C6—C1	−176.7 (3)	C8—C9—C10—O2	−177.9 (3)
C4—C5—C6—C7	−174.5 (4)	C11—C9—C10—C12	−180.0 (4)
N1—C5—C6—C7	6.7 (5)	C8—C9—C10—C12	2.8 (6)
C1—C6—C7—F3	114.9 (4)	O2—N2—C11—C9	1.2 (4)
C5—C6—C7—F3	−68.6 (5)	C10—C9—C11—N2	−0.4 (4)
C1—C6—C7—F2	−5.7 (6)	C8—C9—C11—N2	176.9 (3)
C5—C6—C7—F2	170.9 (4)		

### Hydrogen-bond geometry (Å, º)

**Table:**

*D*—H···*A*	*D*—H	H···*A*	*D*···*A*	*D*—H···*A*
N1—H1*A*···O1^i^	0.86	2.13	2.855 (3)	142

Symmetry code: (i) *x*, −*y*+1/2, *z*+1/2.
